# Artificial Intelligence in Orthopedic Trauma: A Narrative Review

**DOI:** 10.7759/cureus.107574

**Published:** 2026-04-23

**Authors:** Carson Walters, Porter Young

**Affiliations:** 1 Orthopedic Surgery, University of Florida College of Medicine, Gainesville, USA; 2 Orthopedic Surgery, University of Florida-Jacksonville, Jacksonville, USA

**Keywords:** artificial intelligence in orthopaedics, artificial intelligence in surgery, large language models, machine learning in medicine, orthopaedic trauma surgery

## Abstract

Research into the applications of artificial intelligence (AI) within orthopedic trauma has undergone an exponential trajectory. While most studies focus primarily on radiographic fracture detection, the scope of AI has rapidly expanded to include preoperative planning, surgical education, and administrative optimization. This narrative review synthesizes evidence using PubMed, OpenEvidence, and Google Scholar to provide a comprehensive overview of AI’s current utility and its future implications for the field. Current diagnostic applications represent the most established use of AI, with meta-analyses demonstrating that AI-assisted radiographic interpretation achieves a pooled sensitivity and specificity of 87% and 92%, respectively. Beyond 2D imaging, AI has revolutionized surgical preparation by generating patient-specific 3D anatomical models much faster than traditional manual methods. In the realm of surgical education, AI is transitioning resident assessment from subjective evaluation to a quantitative science by providing objective feedback on metrics like economy of motion. However, while AI can assist in manuscript development and research gap analysis, the risk of "hallucinations" and fabricated citations necessitates rigorous human oversight. Other non-surgical applications offer significant potential for enhancing healthcare equity and clinician well-being. AI-assisted diagnostics have been shown to reduce missed fractures, while "AI scribes" can save surgeons up to an hour of documentation daily. However, despite these advancements, a significant "validation gap" remains. Significant hurdles regarding professional liability, the "efficiency paradox" of increased patient workloads, and the need for multicenter testing must be addressed before AI can be fully integrated into the standard of care. Ultimately, AI represents a transformative shift in orthopedic trauma, moving the field toward a future where clinical decision-making and surgical technique are interwoven with AI assistance to improve patient outcomes and physician well-being.

## Introduction and background

In recent years, the volume of literature regarding artificial intelligence (AI) in orthopedic trauma has grown exponentially. While the most common research of machine learning (ML) and deep learning (DL) within the field is in relation to detecting fractures at 24.4%, the potential applications have rapidly expanded to include surgical planning, clinical workflow, and expediting research data [1]. With these technologies becoming increasingly applied to the standard orthopedic trauma workflow, it is imperative to have a foundational understanding of the vast applicational abilities within the field. AI in orthopedic trauma has begun displaying many promising usages, ranging from diagnostic capabilities [1] to enhancing the quality of training for orthopedic residents [2]. However, the accelerated emergence of AI tools has created significant clinical and ethical challenges. While the potential in streamlining work within orthopedic trauma is substantial, physicians should remain cautious with AI usage. Many tools lack significant external validation with diverse patient populations [1]. Generative AI also poses the risk of “hallucinatory” responses, which significantly mislead clinical decision-making and patients [3]. This article provides a comprehensive overview of these multifaceted applications of AI in orthopedic trauma, as well as critically evaluates the current limitations and future implications for the field.

## Review

Methods

A literature search was conducted across OpenEvidence, PubMed, and Google Scholar to identify studies exploring the integration of AI in orthopedic trauma. The search strategy utilized a combination of targeted keywords and Boolean operators, including ("Artificial Intelligence" OR "Large Language Models" OR "Machine Learning") AND ("Orthopedic Trauma" OR "Fracture Detection" OR "Surgical Education" OR "Physician Workflow"). OpenEvidence served as the primary search engine to identify high-yield, peer-reviewed sources and current clinical evidence. The inclusion criteria focused on articles published through February 2026 that demonstrated actionable utility in the orthopedic trauma landscape. To provide a comprehensive clinical framework, the search included "contextual literature" (e.g., addressing surgeon time management, physician burnout, and patient literacy), which served as a baseline to evaluate the potential impact of AI-driven solutions. Articles were excluded if they were unrelated to the musculoskeletal (MSK) system or lacked translatable findings for orthopedic trauma-specific workflows. A total of 112 citations were initially identified and screened for thematic relevance, with 42 articles ultimately selected for synthesis based on their clinical and non-clinical applicability to the orthopedic trauma field.

Diagnostic accuracy and imaging

Fracture Detection and Classification

The most established application of AI within orthopedic trauma is the optimization of radiographic interpretation, specifically regarding fracture identification [1]. In a large-scale meta-analysis involving over 9,000 radiographs, researchers evaluated AI’s ability to assist clinicians in the diagnostic process. The results demonstrated a significant increase in pooled sensitivity and specificity, rising to 87% and 92%, respectively [4]. Importantly, this improvement in accuracy did not come at the cost of clinical time; AI assistance was found to have no significant impact on the reading speed of clinicians, suggesting it serves as an efficient "second opinion" rather than a hurdle to the routine workflow [4]. While AI demonstrated a standalone diagnostic accuracy of 87.29%, its greatest utility appears to be as a collaborative tool that augments clinicians’ expertise [4].

Beyond simple detection, AI is demonstrating significant potential in the more complex task of fracture classification. While identifying a fracture is the first step, correctly classifying its pattern is essential for developing an effective surgical or conservative treatment plan. This is particularly evident in pelvic and hip fractures-common and high-morbidity cases in orthopedic trauma [5]. A study evaluating AI’s ability to classify these specific injuries achieved an accuracy between 94.66% and 97.67%, whereas clinicians demonstrated a mean accuracy of 84.53% on the same studies [6]. By outperforming clinical averages in pattern recognition, these AI models provide a higher level of diagnostic clarity that could directly lead to more precise operative planning and improved patient outcomes.

In the high-pressure environment of trauma activations, the impact of AI on extremity fracture detection is even more pronounced. One study reported a standalone AI accuracy of 98.6%, which facilitated a 27% decrease in the average time required for radiograph reading [7]. This study, which involved eight specialists each from emergency medicine, MSK radiology, and non-MSK radiology, highlights a vital implementation for trauma-related situations where timely data relay to orthopedic trauma surgeons is paramount. Further emphasizing this clinical safety net, another study utilizing 480 radiographs-pre-read by two MSK radiologists-found that AI assistance increased the sensitivity of fracture detection by 10.4% [8]. By significantly reducing the number of false negatives, AI directly addresses the "missed fracture" phenomenon that remains a leading cause of litigation and morbidity in trauma care [9]. The scope of AI-driven diagnostics is rapidly expanding beyond static 2D radiographs. Algorithms have demonstrated comparable efficiency in interpreting CT scans, with an area under the curve (AUC) for X-ray versus CT detection both reaching 0.96 [10]. This multimodal capability suggests that AI can maintain high-fidelity performance across the various imaging stages of a trauma workup.

Anatomical Specificity

A critical takeaway for the practicing surgeon is that AI performance is not uniform. Significant differences in accuracy exist based on the skeletal region being evaluated; for instance, some programs demonstrate superior accuracy on knee radiographs compared to those of the foot, wrist, or hand [11]. This highlights a realistic hurdle for future implementation: a "one-size-fits-all" algorithm is an unlikely future. Instead, future workflows will likely incorporate a "plethora" of specific AI programs, each trained for a distinct anatomical scenario or subspecialty.

This regional variance is further evidenced by a meta-analysis of 100 studies, which found that tibia and fibula fractures were associated with an AI sensitivity 7% higher than other anatomical regions [12]. However, many current FDA-approved products struggle with complex axial or small-bone structures, such as rib fractures, which showed a pooled sensitivity of only 0.66, and spinal injuries, which demonstrated a pooled specificity of 0.63 [13]. However, specialized models continue to emerge, such as an AI program developed specifically for pelvic radiographs that achieved a 90.2% diagnostic accuracy for osteoarthritis with a sensitivity of 97.6% [14].

Research methodologies

Exponential Growth

The volume of AI-related literature in orthopedic trauma has followed an unprecedented exponential trajectory. Highlighting the rapid acceleration of this field, 52.5% of all studies concerning AI in orthopedic trauma were published in the year 2024 alone [1]. This surge reflects a categorical shift in focus in recent years of research.

AI is being leveraged to improve the efficiency of the research process itself, particularly in the synthesis of vast datasets. In one notable methodological application, a ML algorithm was tasked with analyzing 21,968 abstracts related to orthopedic trauma to categorize them into 30 distinct thematic topics [15]. By ranking these topics according to their prevalence in the literature, the AI demonstrated an ability to save researchers an immense amount of time in identifying current trends and, more importantly, pinpointing significant gaps in the existing literature [15]. For a field as broad as trauma, this automated "gap analysis" is essential for directing funding and effort toward under-researched clinical problems.

Generative AI in Manuscript Development

The arrival of large language models (LLMs) has introduced the possibility of automated manuscript generation. Studies testing the ability of LLMs to draft orthopedic journal articles have concluded that these models are capable of producing text that meets the structural requirements of a publishable manuscript [3]. However, this capability is currently tethered to a significant limitation: the prevalence of "hallucinations." LLMs frequently generate or cite information that is entirely fabricated, necessitating extreme scrutiny and manual verification by human authors [3]. While AI can significantly accelerate the ideation and drafting phases of research, it cannot yet replace the critical appraisal and ethical oversight of a human researcher.

The integration of generative AI into the academic workflow of orthopedic trauma is already occurring. Analysis of articles published in “Orthopaedics & Traumatology: Surgery & Research” reveals that in the 10 months following the commercial release of ChatGPT (OpenAI, San Francisco, CA, USA) in November 2022, the utilization of the model in manuscript preparation rose to 15.94% [16]. This represents a notable increase from the 10.30% utilization rate observed in the nine months prior to the model's release, signaling a rapid adoption of LLMs within the orthopedic trauma literature [16]. However, the rise of AI-assisted drafting introduces significant challenges for editorial integrity, as current AI-detection software is not 100% accurate and often fails to provide fully reliable verification of a manuscript's origin [17]. The rise of AI implementation in research literature and the lack of certain verification place a difficult burden on journals to maintain ethical and appropriate policies.

Regulatory and Publication Landscapes

The rapid influx of AI-generated content has outpaced the standardization of editorial policies. An evaluation of 100 orthopedic and sports medicine journals revealed a fragmented regulatory environment; while 82% of journals allowed for AI-generated text and 60% permitted AI-assisted image generation, 6% of journals offered no guidelines regarding AI usage whatsoever [18]. This incongruency in publication standards poses a risk to the integrity of orthopedic literature, as what is considered valid research in one journal may be viewed as non-compliant in another [18]. Establishing universal regulatory guidelines is a crucial next step for the orthopedic academic community to ensure that AI-assisted research maintains a consistent standard of rigor.

Orthopedic training and education

Objective Assessment

In the realm of orthopedic trauma surgical education, AI is transforming the traditionally subjective process of skill assessment into a quantitative science. AI systems are now capable of providing objective feedback on surgical techniques by measuring metrics such as economy of motion and tool path lengths [19]. These parameters provide an unbiased record of a resident’s technical proficiency, allowing programs to track competency and efficiency with a level of precision that human attendings, each with their own subjective habits, cannot consistently provide [19]. This transition toward quantitative measurement facilitates a more equitable evaluation of residents across different training programs.

Beyond the operating room, AI is also demonstrating a sophisticated understanding of orthopedic examinations. In a study utilizing the 2024 Orthopedic In-Training Examination (OITE), 10 out of 11 commercial AI models successfully passed the exam, with the OpenAI model achieving a peak accuracy of 90.8% [20]. However, this accuracy varied by topic. The models performed worst on the topics of trauma and adult hip and knee reconstruction, averaging 71.9% and 71.8%, respectively [20]. Its highest-scoring topic, shoulder and elbow, averaged 91.7% [20]. One study evaluating ChatGPT-4’s ability to answer OITE questions that included images found the model's accuracy dropped to just 49% [21]. This demonstrates that AI can serve as a powerful study assistant; however, its reliability may vary significantly depending on the subspecialty and question style.

Generative AI and the Educational Gap

Despite the potential of LLMs to assist in training, current models face significant limitations in educational content creation. When ChatGPT-4 was evaluated for its ability to produce OITE-quality practice questions, orthopedic educators flagged 70% of the AI-generated questions for containing at least one error [22]. With regard to trauma-specific questions, 19% of the questions were noted to have inaccuracies, but 62.5% of the questions were ranked as having similar difficulty to the real exam. This suggests that while LLMs are proficient at answering questions, they are not yet reliable for creating them [22]. In evaluating an AI's ability to answer commonly asked orthopedic trauma questions, the system achieved a 70.5% correctness rate when addressing an expert orthopedic surgeon, but this dropped significantly to 52.9% when explaining the same concepts at a patient’s level [23]. These results suggest that while AI is increasingly effective as an expert clinical resource, substantial improvements are still required to ensure accurate and reliable patient education within the trauma field [23].

Furthermore, AI's predictive capabilities are being utilized to identify educational gaps before they manifest as failed board exams. By analyzing a resident’s performance on internal exams and intraoperative assessments, AI can project future board exam performance with high accuracy [24]. This allows residency programs to be proactive rather than reactive, providing targeted interventions for residents who may be struggling to meet the necessary benchmarks for certification [24].

Simulation and Virtual Reality

The integration of AI into virtual reality (VR) and augmented reality (AR) simulators allows residents to practice high-acuity trauma procedures in a risk-free environment. Unlike traditional simulators, AI-driven platforms can generate complex, non-routine surgical scenarios that a resident might not encounter during a standard rotation [25]. Evidence suggests that residents utilizing these simulators show marked improvements in speed, accuracy, and clinical confidence compared to control groups [20]. For orthopedic trauma, a field defined by high-stress, time-sensitive decision-making, the ability to "fail" and learn in a simulated environment is a critical component of developing surgical maturity before entering the operating room.

Non-surgical applications

Health Equity

AI facilitates access to more equitable care in rural and resource-limited settings by providing critical diagnostic support to clinicians with limited MSK imaging training. Implementation of AI-assisted diagnostics has demonstrated a 55% reduction in missed fractures, lowering the diagnostic error rate from 20% to 9% [26]. This enhanced accuracy at the point of entry will help ensure timely subspecialty referral and possibly minimize complications associated with delayed fracture management, thereby optimizing the entire orthopedic trauma treatment pathway.

Furthermore, AI is being leveraged to bridge the communication gap between surgeons and their patients. Currently, patient education materials in many hospital libraries are written at a level far above the national sixth-grade readability guidelines [27]. Using LLMs to simplify these documents successfully reduced the average reading level of 806 orthopedic patient education documents to a grade of 6.1 [27]. This explainable AI ensures that patients have a clear understanding of their treatment plans and outcomes, which is a vital component of postoperative recovery and satisfaction.

Decreasing Trauma Workload

The acuity and volume of orthopedic trauma consultations can be challenging to manage. AI-driven tools implemented for limb trauma in the emergency department (ED) have shown the potential to reduce consultation rates, allowing surgeons to focus on higher acuity cases [28]. The orthopedic trauma consultation rate was 6.3% in the AI-driven group versus 7.1% in the control group [28]. While this finding was not statistically significant in initial studies, it demonstrates the potential for reducing the burden on orthopedic trauma physicians [28].

The implementation of AI in limb trauma workflows has, however, resulted in a significant nine-minute reduction in a patient’s total time spent in the ED [28]. While this requires further evaluation on a larger scale to determine meaningful clinical significance, it suggests a path toward lowering the workload burden of trauma surgeons. Reducing unnecessary time spent in the ED has the downstream effect of increasing overall productivity and hospital workflow.

Administrative Streamlining

The emergence of "AI scribes" represents a major advancement in clinical workflow by listening to physician-patient conversations and writing the physician’s note in real time [29]. On average, physicians utilizing this technology spend 20% less time on screens and save up to an hour of documentation daily [29]. Given that orthopedic surgeons see an average of 31 patients per clinic day, this is a crucial time spent [30]. Currently, some orthopedic specialists spend an average of 7.2 minutes on the electronic health record (EHR) per visit, accounting for 36% of the total encounter [31]. By implementing AI scribes, this number can be significantly reduced to give more attention to the patient, which has been shown to correlate with higher overall patient satisfaction [32].

Preoperative planning and 3D modeling

Modern AI has revolutionized preoperative preparation by transforming standard cross-sectional imaging into patient-specific anatomical maps. Algorithms can now generate unique 3D models using CT and MRI scans, providing surgeons with a visualization of complex fracture patterns that exceeds the clarity of traditional 2D imaging [33]. This increased spatial awareness is critical for a successful operative strategy in difficult orthopedic trauma scenarios. Furthermore, AI models have demonstrated the ability to produce bone models in an average of only 233 s compared to traditional 3D generation, which can require up to 30 minutes [34].

Surgical application

Intraoperative Guidance

The emergence of "digital twin" technology and AI-assisted guidance has shown direct benefits in operating room efficiency. The "digital twin" concept represents a high-fidelity AI simulation that mirrors a patient’s specific anatomical and physiological state in real time, providing both live diagnostic data and predictive clinical outcomes [35]. In orthopedic trauma revision surgery, where the clinical goal is to achieve a result superior to the failed initial intervention, this technology allows the surgical team to visualize the prospective mechanical consequences of a revision strategy before it is permanently executed. By utilizing these simulations, surgeons can make precise intraoperative adjustments to optimize fracture strain and implant stress distribution, ensuring the procedure provides the most stable biomechanical environment possible [35]. As these technologies are adapted for orthopedic trauma, the gains in both operative speed and long-term functional recovery are expected to be substantial.

Postoperative Monitoring

Postoperative care, particularly the management of surgical site infections (SSIs), remains a significant challenge in trauma. SSI prevalence can reach 19% in high-risk closed injuries and over 30% in severe open fractures [36]. To address this, ML software has been developed to predict SSI occurrence in lower extremity fractures by analyzing predictors such as body mass index (BMI), age, operative time, and injury location [37]. These models have achieved an AUC of 77.8%, allowing surgeons the potential opportunity to implement personalized prophylactic measures [37]. This has the potential to reduce significant adverse effects caused by SSIs, including hospital stays and overall mortality.

Future directions and limitations

Addressing the Validation and Implementation Gap

One of the most significant barriers to the widespread clinical adoption of AI is the current "validation gap." Despite the surge in orthopedic AI research, only 14.5% of these studies have undergone external validation [1]. This lack of external testing leaves a substantial void in the literature, as algorithms trained on localized datasets may harbor inherent population biases that render them inaccurate when applied to different demographics or hospital systems [1].

Furthermore, many current AI models exist within "closed systems," meaning they are trained using data from a single institution [38]. While these algorithms may demonstrate high efficiency within their original environment, they often lack the generalizability required for broad clinical application [38]. For AI to become a reliable tool in orthopedic trauma, future research must prioritize multicenter validation and the development of "open-system" models that can maintain accuracy across diverse clinical settings.

The Dilemma of Liability and Training

A critical, unresolved issue is the question of legal and professional liability. In high-stakes trauma scenarios-such as triage-if an AI incorrectly classifies a patient’s acuity, resulting in a suboptimal clinical outcome, it remains unclear where the fault lies [38]. This ambiguity creates a significant "hesitancy barrier" for hospitals and physicians. Until government intervention or regulatory bodies establish a clear legal framework for AI-related clinical faults, many systems will likely remain simply a proof-of-concept [38].

Additionally, the integration of AI will necessitate a fundamental change in medical education. As AI begins to generate complex diagnoses and treatment plans, surgeons will require additional training to critically evaluate the efficacy of these machine-generated proposals [39]. This creates a new cognitive burden, particularly in the trauma subspecialty, where surgeons must often make split-second decisions with incomplete data. The "human-in-the-loop" model requires the physician to be not only a master of surgery but also a competent overseer of the algorithms they employ [39].

Clinical Burnout

There is also a significant concern regarding the "efficiency paradox." While AI is designed to streamline administrative tasks, this newfound efficiency may create an unwarranted expectation for physicians to increase their patient volume. For orthopedic trauma surgeons, who already manage higher-than-average patient loads, this pressure could exacerbate physician burnout rather than alleviate it [39]. The successful integration of AI must therefore be balanced with institutional policies that prioritize surgeon well-being alongside technological productivity.

Increasingly Autonomous Care

A future advancement in AI is the move toward multimodal integration. This represents processing radiographic imaging, EHR, and intraoperative data simultaneously [40]. By synthesizing these streams, AI develops a holistic, case-specific understanding that accounts for unique biological and logistical variables [40]. This precision can significantly reduce adverse care decisions arising from incomplete clinical history reviews in emergent cases [40]. Looking ahead, AI-facilitated smart examination rooms could utilize integrated sensors to gather objective data, such as gait analysis or range of motion, providing a level of diagnostic granularity that exceeds manual examinations [41].

More immediate transformations involve AI agents that perform digital tasks autonomously [40]. In trauma settings, these agents could revolutionize operating room scheduling by automatically adjusting for emergencies, mitigating overbooking and triage errors [40]. Furthermore, agents could alleviate administrative burdens, given that orthopedists spend 58% of their day on EHR tasks, by optimizing follow-up schedules or note writing in the background [30,40]. Academically, autonomous agents could continuously synthesize new literature to identify data gaps, ensuring trauma orthopedists remain at the forefront of evidence-based medicine without manual searching (Table 1) [42].

**Table 1:** Summary of AI Applications in Orthopedic Trauma Summary of original research evaluating AI applications within orthopedic trauma surgery. AI: artificial intelligence; OITE: Orthopedic In-Training Examination; ED: emergency department

Reference	AI model/technology	Sample size/scope	Key trauma-specific findings
Yilmaz et al. [6]	Automated deep learning	326 pelvic radiographs	Achieved 94.66%–97.67% accuracy for hip fracture classification
Rowley et al. [15]	Machine learning	21,968 trauma abstracts	Categorized literature into 30 thematic topics to identify data gaps
Bisi et al. [16]	AI-output detectors	425 Orthopaedics & Traumatology articles	AI use in manuscripts rose from 10.30% to 15.64% post-ChatGPT
Hayes et al. [20]	ChatGPT-4	119 OITE text-based questions	90.8% peak accuracy; models performed lowest in trauma (71.9%)
Dagher et al. [22]	ChatGPT-4	20 AI-generated OITE practice questions	70% of AI questions flagged for containing at least one error, trauma included
Kaarre et al. [23]	ChatGPT-4	34 AI-generated trauma surgery question responses	Correctness was 70.5% for experts but only 52.9% for patients
Herpe et al. [28]	AI limb software	3,720 emergency room patients with an orthopedic trauma	9-minute reduction in ED time; consult rate fell from 7.1% to 6.3%
Andres et al. [35]	Digital twin technology	5 orthopedic trauma cases	Mirrors physiology to optimize fracture strain and implant stress
Bignami et al. [38]	Ethical frameworks	Trauma care integration	Highlights unresolved liability dilemmas for AI triage errors

## Conclusions

AI is increasingly redefining the paradigms of orthopedic trauma management and education. Despite these advancements, the transition to widespread clinical usage is restricted by a lack of external validation and complex implementation challenges. Future progress in the subspecialty depends on establishing a clear framework for liability and ensuring these algorithms are robust across diverse clinical settings.
